# The impact of chronic fatigue syndrome on cognitive functioning in adolescents

**DOI:** 10.1007/s00431-015-2626-1

**Published:** 2015-09-03

**Authors:** Linde N. Nijhof, Sanne L. Nijhof, Gijs Bleijenberg, Rebecca K. Stellato, Jan L. L. Kimpen, Hilleke E. Hulshoff Pol, Elise M. van de Putte

**Affiliations:** Department of Pediatrics, Wilhelmina Children’s Hospital, University Medical Centre Utrecht, HP KE.04.133.1, Post box 85090, 3508 AB Utrecht, The Netherlands; Expert Center Chronic Fatigue, Radboud University Nijmegen Medical Centre, Nijmegen, The Netherlands; Biostatistics, Julius Center for Health Sciences and Primary Care, University Medical Centre Utrecht, Utrecht, The Netherlands; Rudolf Magnus Institute of Neuroscience, Department of Psychiatry, University Medical Centre Utrecht, Utrecht, The Netherlands

**Keywords:** Adolescent, School performance, Chronic fatigue syndrome, Cognitive impairment, IQ

## Abstract

Chronic fatigue syndrome (CFS) is characterized by persistent fatigue and severe disability. Most adolescent patients report attention and concentration problems, with subsequent poor performance at school. This study investigated the impact of CFS on intellectual capacity by (1) assessing discrepancies between current intelligence quotient (IQ) and school level and (2) exploring differences in current IQ and pre-CFS school performance, compared with healthy individuals. Current data was cross-sectionally gathered and compared with retrospective pre-CFS school performance data. Fifty-nine CFS adolescents and 40 controls were evaluated on performance on age-appropriate intelligence tests and school level. Current IQ scores of CFS adolescents were lower than expected on the basis of their school level. Furthermore, there was a difference in intelligence performance across time when current IQ scores were compared with pre-CFS cognitive achievement. Healthy controls did not show any discrepancies.

*Conclusion*: According to their pre-CFS intelligence assessments, CFS patients started with appropriate secondary school levels at the age of 12. Our data suggest that CFS may be accompanied by a decline in general cognitive functioning. Given the critical age for intellectual development, we recommend a timely diagnosis followed by appropriate treatment of CFS in adolescents.

**Table:** 

**What is Known:** • Adolescent chronic fatigue syndrome (CFS) is a debilitating condition with major impact on social and intellectual development. • Most patients report concentration problems, with subsequent poor performance at school. Little is known about the influence of CFS on intellectual performances.
**What is New:** • IQ scores of CFS adolescents are lower than the IQ scores of healthy peers with an equivalent school level. • There is a decrease in intelligence performance across time when current IQ scores are compared with pre-CFS cognitive achievement. Healthy controls do not show any discrepancies between their current IQ, school level and previous cognitive functioning. This suggest that adolescent CFS may be accompanied by a decline in general cognitive functioning.

## Introduction

Chronic fatigue syndrome (CFS) in adolescents is a disabling condition characterized by severe and unexplained fatigue lasting for more than 6 months, with long-term consequences for educational and social development [6, 20]. The fatigue is accompanied by additional symptoms such as headaches, myalgia, multiple joint pain, unrefreshing sleep, and memory and concentration problems. Somatic and psychiatric illnesses must be excluded to meet the criteria for CFS [11].

The prevalence of CFS in adolescents has been estimated at between 0.11 and 1.29 % in community studies in the USA and Europe, with a female to male ratio up to 5:1 [3, 10, 17, 25, 29].

Both adult and adolescent CFS patients frequently report cognitive problems associated with a perceived change in intellectual abilities [21]. Memory and concentration problems are reported by, respectively, 89 % of adults [16] and 80 % of adolescents [7]. Several studies, mainly focused on adults, have described impaired cognitive performance by CFS patients on neuropsychological tests. These tests measured information-processing speed, memory, motor speed and executive functioning [5]. Reduced speed of complex information processing is the impairment most consistently found [5, 21, 22, 32].

Our clinical observations confirm that CFS adolescents experience a change in cognitive functioning with subsequent poor performance at school. This often leads to a drop in school level during CFS, with subsequent feelings of frustration.

In contrast to memory and concentration problems in CFS patients, the intellectual capacity in adolescents with CFS has rarely been investigated. One study measured the discrepancy between actual and perceived intelligence quotient (IQ) by parents of adolescents with CFS compared to those of parents of healthy peers in a cross-sectional design [12]. Parental expectations of IQ were significantly higher than the actual IQ for children with CFS. The authors concluded that high parental expectations might contribute to the development of CFS and that high expectations may need to be addressed within the context of treatment. However, because of the cross-sectional study design, it remained unclear whether these discrepancies in IQ were causal factors or consequences of CFS.

In this study, we explore the hypothesis that adolescents with CFS show a discrepancy between current IQ and school level irrespective of psychological functioning. Firstly, we compare actual IQ levels of CFS adolescents with those of healthy controls of the same school level. Secondly, we extend the comparison with pre-secondary school IQ levels in both groups. These two comparisons enable us to explore the question if a current difference in IQ level was already apparent in the pre-CFS phase (i.e. pre-secondary school).

## Materials and methods

### Design and population

In this study, current IQ data were cross-sectionally gathered and compared with retrospective standardized school performance assessments at primary school (CITO). This longitudinal design was used to compare intellectual performance in adolescents with CFS and healthy adolescents. Between August 2009 and February 2010, 64 adolescents (12–18 years) participating in the FITNET (Fatigue in Teenagers on the Internet) trial [23, 24] were invited to participate in this neurocognitive study when attending their initial assessment at the University Medical Centre Utrecht. Fifty-nine patients agreed to participate (92 %). All patients met the criteria for chronic fatigue syndrome as defined by the Centers for Disease Control and Prevention (CDC), Atlanta, GA, USA [11]. Exclusion criteria were primary depression, anxiety disorder or suicidal risk [23].

As a reference group, healthy participants from a former control group were re-invited between August 2009 and February 2010 [25]. From this group, adolescents with neurological abnormalities, chronic illnesses or under treatment from a psychiatrist or psychologist were excluded from participation. Forty of 58 eligible healthy adolescents (69 %) completed age-appropriate intelligence tests. In view of the demographic characteristics and fatigue levels, both the CFS-patients and controls were representative samples of former studies [23, 25].

### Outcome measures

1. Intelligence. All subjects completed age-appropriate short versions of the Wechsler intelligence scales, 3rd edition, Dutch version, to determine intelligence (WISC-III-NL for adolescents until 16 years old and WAIS-III-NL for adolescents of 17 and 18 years old). Full-scale IQ was estimated from four subtests (picture completion, information, block design and vocabulary) [13, 19, 30, 31]. Cronbach’s alpha for the short version is 0.91 and for the full test 0.95 [13]. IQ was corrected for age and sex.
2. CITO. Cognitive abilities and educational achievement in primary school were assessed using the Dutch CITO elementary test (Centraal Instituut Toets Ontwikkeling, www.cito.com). The CITO consists of 240 multiple-choice items assessing four different intellectual skills: language, mathematics, information processing and world orientation. Taken together, the performance scales result in a standardized score between 501 and 550. The test is usually administered on three consecutive days in January or February when the children are in the final grade of primary school and approximately 12 years old. This assessment is not a formal IQ test but highly correlates with IQ performance (correlation of 0.63 between CITO and IQ assessed at age 12) [1] and plays an important advisory role in the choice of secondary school education [27]. In the Netherlands, everyone receives comparable primary education from 4 to 12 years of age. Thereafter, depending on general performance and assessment of intellectual capacity measured by the CITO test, a child follows one of four levels of secondary education (VMBO-basis, VMBO-theoretical, HAVO and VWO, corresponding respectively to ‘below average’, ‘average’, ‘above average’ and ‘high’ levels of attainment). In this study, we have recruited only participants (patients and healthy controls) from the last three categories. The general availability of CITO test scores makes it possible to compare current cross-sectional data with retrospective intelligence data.
  Copies of the official CITO reports were collected by email from the adolescents themselves or from teachers after informed consent was obtained. Forty-nine CFS patients and 34 healthy controls (83 % resp., 85 % response) supplied a copy of their official CITO scores. None of these adolescents had symptoms of CFS at the time of completing the CITO test.

### Psychological functioning

Self-reported questionnaires were used to assess fatigue, concentration problems, anxiety, depression and achievement motivation. Participants were asked to fill out these questionnaires without assistance from their parent(s) or the researcher.

Fatigue and concentration problems were assessed using the self-report questionnaire checklist individual strength (CIS-20), subscales ‘fatigue severity’ (8 items) and ‘concentration problems’ (5 items). The CIS-20 is a reliable assessment tool with excellent internal consistency (Cronbach’s α 0.93) and discriminative validity for CFS [33].

Assessment of trait anxiety in adolescents was performed with a validated Dutch translation of the Spielberger State-Trait Anxiety Inventory for Children (STAIC) [26]. The STAIC consists of 20 statements on a three-point scale.

Depression in adolescents was measured using a validated Dutch translation of the Children’s Depression Inventory (CDI) [18]. The CDI quantifies depressive symptoms from the past 2 weeks and consists of 27 items rated on a three-point scale.

Fear of failure was determined in the adolescents by means of the Achievement Motivation Test for Children (PMTK), a validated Dutch questionnaire that expresses performance pressure, negative fear of failure, positive fear of failure, socially desirable behaviour and combined fear of failure in deciles (1–10) [15].

### Ethics

The medical ethics committee of the University Medical Centre Utrecht approved the study. Written informed consent was obtained from all adolescents and their parent(s), according to the Declaration of Helsinki.

### Data analysis

All variables were tested for normality and if normality was confirmed, parametric statistics were used. Differences between groups were assessed using the independent sample *t* test and *χ*^2^ test. We divided the school educational levels into three groups: level 1 (VMBO-theoretical; average), level 2 (HAVO; above average) and level 3 (VWO; high level).

A two-way ANOVA model was used to explore a possible discrepancy between IQ and school level for CFS patients and healthy controls; the dependent variable was the IQ score and the independent variables were the group (CFS yes/no), the current school level and their interaction.

To examine the effect of CFS on IQ while correcting for potential confounders, linear regression models with CFS (yes/no), age, gender and initial school level were used. Statistical significance was assumed at *p* < 0.05. Statistical analyses were performed using IBM SPSS Statistics 20.

## Results

### Descriptive statistics

The baseline characteristics of the participating adolescents (59 CFS patients and 40 healthy controls) and those who did not want to participate (5 CFS patients and 18 healthy controls) did not differ (data not shown).

Table 1 compares the demographic characteristics and psychological functioning of CFS patients with healthy adolescents. The median duration of CFS inpatients was 20.0 months. Girls predominated both groups, but were significantly overrepresented in the CFS group. Adolescents with CFS reported more fatigue and concentration problems than healthy controls. Also, anxiety and depression levels were higher in the CFS group. There was no difference in achievement motivation.

**Table 1:** Demographic features of adolescents with CFS and healthy adolescents

	CFS^a^ *n* = 59	Healthy^a^ *n* = 40	Mean difference (95 % CI)	*p* value
Demographic characteristics				
Gender—% girls	78	55	–	0.026
Age at entry—years	15.8 (1.5)	15.7 (0.5)	−0.2 (−0.6; 0.2)	0.394
Duration of CFS symptoms at entry—months^£^	20.0 (17.0)	–	–	–
School absence—%	52.9 (33.0)	1.5 (2.8)	51.3 (42.7; 60.0)	<0.001
Fatigue assessment				
CIS score severity of fatigue (8–56)	51.5 (4.4)	21.2 (9.9)	30.3 (26.9; 33.6)	<0.001
CIS score concentration problems (5–35)	29.3 (5.6)	16.0 (6.2)	13.3 (10.9; 15.7)	<0.001
Psychological adjustment				
Anxiety disposition (STAIC; 20 items; 20–60)	32.3 (7.7)	27.9 (6.6)	4.3 (1.3; 7.3)	0.005
Depression disposition (CDI; 27 items; 0–54)	11.4 (5.7)	5.8 (4.0)	5.6 (3.5; 7.6)	<0.001
Achievement motivation				
PMT-K need to perform	4.6 (2.5)	4.1 (2,4)	0.6 (−0.4; 1.6)	0.268
PMT-K negative fear of failure	4.4 (2.7)	3.9 (2.6)	0.5 (−0.6; 1.6)	0.377

*CFS* chronic fatigue syndrome, *CI* confidence interval, *CIS* checklist individual strength, *STAIC* State-Trait Anxiety Inventory for Children, *CDI* Children’s Depression Inventory, *PMT-K* Achievement Motivation Test for Children

^a^Values are means (SD) unless otherwise stated

^£^Median (IQR)

Table 2 shows school levels and intelligence assessment scores of adolescents with CFS and healthy adolescents. There was no statistically significant difference between CFS patients and the controls regarding initial school level (first-grade secondary school) and current school level (current grade). Notably, 9 of 59 CFS patients (15.3 %) already switched to a lower educational level during the course of the disease. This is in contrast to healthy peers who remained at the same level or switched to a higher (*n* = 2) school level. A decision to switch school level is made in commission by the teachers, parents and the student based on (lack of) academic performance.

**Table 2:** Intelligence assessment scores of adolescents with CFS and healthy adolescents

	CFS (SD)^a^ *n* = 59	Healthy (SD)^a^ *n* = 40	Mean difference (95 % CI)	*p* value	Adjusted difference (95 % CI)^b^	*p* value
CITO score^c^ (501–550)	540.4 (7.0)	541.0 (6.6)	−0.6 (−3.6; 2.4)	0.705	−1.0 (−3.1; 1.1)	0.329
Initial secondary school level^d^						
Level 1—%	28.8	22.5				
Level 2—%	28.8	35.0		0.721		
Level 3—%	42.4	42.5				
Current secondary school level						
Level 1—%	32.2	22.5				
Level 2—%	37.3	30.0		0.223		
Level 3—%	30.5	47.5				
Intelligence level						
Measured IQ score^e^	103.3 (11.4)	111.2 (11.0)	−8.0 (12.6; −3.3)	0.001	−6.5 (−10.9; −2.1)	0.004
Performance IQ						
Block design (1–18)	10.3 (2.5)	11.8 (2.2)	1.5 (0.6; 2.5)	0.002	−1.4 (−2.4; −0.4)	0.006
Picture completion (1–18)	9.1 (2.9)	10.7 (2.1)	1.6 (0.5; 2.7)	0.005	−1.3 (−2.5; −0.1)	0.036
Verbal IQ						
Information (1–19)	11.0 (2.8)	11.7 (3.0)	0.7 (−0.6; 2.0)	0.268	−0.3 (−1.5; 1.0)	0.663
Vocabulary (1–19)	11.1 (2.2)	11.3 (2.2)	0.2 (−0.8; 1.1)	0.728	−0.3 (−1.2; 0.7)	0.572

*CFS* chronic fatigue syndrome, *CI* confidence interval, *CITO* Centraal Instituut Toets Ontwikkeling, *IQ* intelligence quotient. IQ: CFS *n* = 59, controls *n* = 39 (1 participant incomplete due to illness)

^a^Values are means (SD) unless otherwise stated

^b^Adjusted for age, sex and initial school level

^c^Intelligence index at the last year of the primary school

^d^Level 1, level 2 and level 3 correspond, respectively, to ‘average’, ‘above average’ and ‘high’ levels of attainment

^e^Estimated IQ measure by subtests

Before disease onset, the CFS patients and the healthy peers did not differ in CITO scores. In contrast, current IQ was 8 points lower for CFS patients than for their healthy peers. Performal IQ contributed most to this difference in IQ since patients performed significantly worse on performance IQ than controls, while no significant difference was found for verbal IQ. Table 2 shows adjusted and non-adjusted differences. The model for adjustment was based on Pearson’s correlations between IQ and school absence (CFS: *r* = 0.15, *p* = 0.25; controls: *r* = 0.25, *p* = 0.13) and between IQ and psychological adjustment (depression—CDI (CFS: *r* = −0.06, *p* = 0.68; controls: *r* = −0.17, *p* = 0.32), anxiety—STAIC (CFS: *r* = 0.14, *p* = 0.31; controls: *r* = −0.22, *p* = 0.19), negative fear of failure (CFS: *r* = −0.12, *p* = 0.39; controls: *r* = −0.18, *p* = 0.27)). Because all correlations were weak, and none was statistically significant, we decided to eliminate these potential confounders from the adjusted model. The differences in IQ are only adjusted for age, gender and initial secondary school level in order to ensure that the difference in current intellectual performance is not explained by a confounding variable alone or to some interaction between those variables.

### Match between school level and intelligence level (IQ)

The mean IQ scores per school level for both groups are shown in Table 3. Adolescents with CFS had lower mean IQ scores than the controls at all school levels. The main effect of CFS was significant (*p* = 0.005), as was the main effect of school level (*p* = 0.001). There was no significant interaction between group (CFS yes/no) and school level (*p* = 0.960).

**Table 3:** Mean IQ score by school level of adolescents with CFS compared with healthy peers

	Educational level 1 (VMBO)^c^	*n*	Educational level 2 (HAVO)^c^	*n*	Educational level 3 (VWO)^c^	*n*
Intelligence quotient (IQ)^a^						
CFS	96.8 (8.6)	19	104.8 (9.2)	22	108.2 (13.5)	18
Controls^b^	104.1 (14.0)	8	110.5 (6.9)	12	114.7 (10.8)	19

There are significant differences between groups at all school levels, *p* = 0.005

*VMBO* Voorbereidend Middelbaar Beroeps Onderwijs, *HAVO* Hoger Algemeen Voortgezet Onderwijs, *VWO* Voorbereidend Wetenschappelijk Onderwijs, *CFS* chronic fatigue syndrome

^a^Values are means (SD)

^b^CFS *n* = 59, Controls *n* = 39 (1 participant incomplete due to illness)

^c^VMBO, HAVO and VWO correspond, respectively, to ‘average’, ‘above average’ and ‘high’ levels of attainment

There was an unadjusted difference of nearly 8 points in IQ score, which persisted after adjustment for age, gender and initial school level (mean difference −6.5; 95 % CI −10.9 to −2.1) as shown in Table 2. No difference in CITO scores after adjustment was found between patients and controls. Also, adjustment for age, gender, initial school level, as well current IQ had no significant effect on estimated CITO test outcomes (mean difference −1.0; 95 % CI −3.7 to 1.8).

## Discussion

In our study, we explored pre-CFS and actual intellectual performance in adolescents with CFS compared with healthy peers. We found that current IQ scores of CFS adolescents were lower than the IQ scores of healthy peers with an equivalent school level. Furthermore, there was a diminishing on cognitive functioning across time when current IQ scores were compared with pre-CFS cognitive achievement. Importantly, CITO scores of CFS adolescents corresponded to their initial secondary school level, as was the case with their healthy controls. Healthy controls did not show any discrepancies between their current IQ, school level and previous CITO score.

The role of IQ in adults and adolescents with CFS has not been well studied. Godfrey et al. studied IQ in CFS adolescents in a cross-sectional study design [12]. Taking into account differences in methodology, our study agrees with Godfrey in that actual IQ in CFS adolescents does not match expectations, which in our study were based on their present school level and pre-CFS school performance. Godfrey et al. showed that parents’ expectations regarding the IQ of adolescents with CFS were significantly higher than those of healthy controls. They concluded that the high expectations of parents could contribute to the development of CFS. However, pre-CFS IQ levels of CFS adolescents in our study were equal to that of healthy peers, suggesting that the actual lower IQ levels in CFS adolescents represent a consequence rather than a cause of CFS. This could imply that the parents in the study of Godfrey et al. had realistic expectations about their children’s intellectual potential but that IQ was affected during the course of the disease.

Many explanations for the lower actual IQ can be postulated. CFS is often accompanied by considerable school absence. The discrepancy between IQ and school level could be explained by the high level of school absence, which leads to reduced knowledge. However, an IQ test should be independent of school attendance, and in this study, we observed no correlation between IQ and school absence in neither the CFS nor in the control group. We therefore postulate that IQ is affected in CFS adolescents independently of absence from school. Also, no correlations between IQ and fear of failure, anxiety or depression were found. These findings are in accordance with the results of Cockshell et al. (2010), who undertook a meta-analysis on the cognitive performance of adults (>16) with CFS compared with healthy controls. Problems in neuropsychological functioning were found to be unrelated to depression, fatigue or anxiety [5]. Thus, neither school absence nor psychological functioning seems to be an adequate explanation for our findings.

Secondly, a (subjective) neurocognitive impairment is inherent in CFS adolescents and might influence the IQ score. Previous studies, mostly in adults, showed a diminished performance in information-processing speed and in tasks requiring working memory over a sustained period of time [4, 5, 21, 22, 32]. In addition, a study specifically in CFS adolescents noted cognitive problems among attention and memory abilities [14]. This could imply that CFS adolescents achieve a lowered IQ score on subtests that require fast processing. The fact that performance intelligence is particularly diminished in our study would fit this premise. If this assumption is true, IQ scores should normalize after treatment of and recovery from CFS.

Thirdly, CFS may affect brain development and thereby cognitive development in adolescence and thus have a possible relationship with a reduced IQ since adolescence is a critical phase of brain development [28]. There are functional magnetic studies (fMRI) that show that activation of the lateral prefrontal cortex is common for a range of intelligence tests and that the magnitude of frontal cortical activation correlates highly with intelligence [2, 31]. Remarkably, MRI studies in CFS patients of de Lange et al. detected lower cortical grey matter volume in adult CFS compared with healthy controls, with a significant increase in grey matter volume in CFS patients, localized in the lateral prefrontal cortex after effective treatment with CBT [8, 9]. This change in cerebral volume was also related to improvements in cognitive speed in the CFS patients. De Lange et al. (2008) conclude that cortical plasticity in the adult human brain is responsible for the change in cerebral volume, demonstrating a surprisingly dynamic relation between behavioural state and cerebral anatomy. Given this possibility, CFS may affect brain development in adolescence and thus have a possible causal relationship with a reduced IQ.

An alternative explanation could be that adolescent CFS patients ‘grow into the deficit’. Although the CITO test highly correlates with IQ scores assessed at the age of 12 [1], it is not a formal IQ test. The CFS adolescents in our study underperformed in particular in the performance tasks, while no significant difference was found for verbal IQ between CFS and healthy adolescents. We cannot exclude a pre-existent difference in performance and verbal IQ (which may not be measured with a CITO test), representing a vulnerability factor, partly responsible for the development of CFS in adolescents.

Some issues need further consideration. We used CITO scores as a pre-CFS proxy of IQ-scores, while the CITO test is not a formal IQ test. However, it highly correlates with IQ scores assessed at the age of 12 [1]. Moreover, the unique setting in the Netherlands, with a general availability of CITO test scores, makes it possible to compare current cross-sectional data with retrospectively collected pre-CFS intelligence data.

The controls were comparable to patients in terms of age and school level, but not for gender. We adjusted for these differences in our analysis.

A strong aspect of our study is the longitudinal design; by assessing pre-CFS intellectual performance, we have two time points, enabling to compare intellectual performance in CFS adolescents and healthy controls in time. Another strength of our study was the high participation rate: 92 % of the eligible CFS patients entered the study, thus reducing the risk of bias. Accurately diagnosing CFS is complex and requires exclusion of other illnesses that could cause similar complaints, but require different treatment. In the FITNET study, CFS was strictly diagnosed using the CDC criteria, in a tertiary academic hospital setting. Since referrals were obtained nationwide and from various sources (general practitioners as well as paediatricians), we consider our study population representative of the Dutch CFS population at large [23]. The control group was recruited for a number of sub-studies of the FITNET study, thus not specifically for a neurocognitive assessment or intelligence test, also reducing the risk of selection bias. In addition, the demographic data indicate that both groups are similar in terms of mean school level and in mean CITO scores before the onset of CFS.

In conclusion, adolescent CFS may be accompanied by a decline in general cognitive functioning. Regardless of the etiological role of intelligence in the emergence of CFS, we advise caution in interpreting intellectual performance tests measured during the course of the disease. It is currently not known whether lower IQ outcomes are due to concentration problems, a lowered processing speed or CFS itself. Given the critical age for intellectual development, we recommend a timely diagnosis followed by appropriate treatment of CFS in adolescents.

## Abbreviations

CBT: Cognitive behavioural therapy
CDC: Centre for disease control and prevention
CDI: Children’s depression inventory
CIS-20: Checklist individual strength
CITO: Centraal instituut toets ontwikkeling
CFS: Chronic fatigue syndrome
CHQ-CF87: Health questionnaire-child form
HAVO: Hoger algemeen voortgezet onderwijs (secondary school of general educational development)
IQ: Intelligence quotient
PMTK: Achievement motivation test for children
SD: Standard deviation
STAIC: Spielberger state-trait anxiety inventory for children
VMBO: Voorbereidend middelbaar beroeps onderwijs (secondary school of general educational development)
VWO: Voorbereidend wetenschappelijk onderwijs (pre-university level)
